# O1.2 PERIPHERAL INFLAMMATORY MARKERS ARE PREDICTIVE OF CLINICAL CHARACTERISTICS AND OUTCOME IN PSYCHOSIS

**DOI:** 10.1093/schbul/sby015.184

**Published:** 2018-04-01

**Authors:** Graham Blackman, Thomas Pollak, Megan Pritchard, John Hanrahan, Anthony Dalrymple, Amalia Velarde, Vivienne Curtis, Robert Stewart, Anthony David

**Affiliations:** 1 Institute of Psychiatry, Psychology and Neuroscience, King’s College London; 2 King’s College London; 3 Ramón y Cajal University Hospital

## Abstract

**Background:**

Dysregulation of the immune system represents an important vulnerability factor for schizophrenia. A rise in peripheral inflammatory markers has previously been demonstrated in psychosis; however, its significance remains uncertain. Characterising this relationship aids our understanding of the role of immunological factors in psychosis, as well as potentially identifying candidate biomarkers to guide diagnosis, treatment and prognosis. Whilst specialized inflammatory marker assays have been found to be associated with outcome and treatment, these tests are not typically available in clinical practice. We sought to establish whether routine inflammatory markers are associated with clinical characteristics and outcomes in patients with schizophrenia and related disorders.

**Methods:**

A multi-site cohort study of patients admitted to an acute psychiatric ward between January 2013 and December 2016 within a large Mental Health Trust was undertaken. Cases were identified from an electronic database containing full clinical records. Inclusion criteria were patients aged 18 and 65 years with a discharge diagnosis of schizophrenia, or related disorder and a routine blood test within 3 days of admission. Exclusion criteria were diagnoses of drug-induced psychosis, organic brain disorder, or admission during the perinatal period. Pro-inflammatory (white blood cell total and differential count, C-reactive protein) and anti-inflammatory markers (albumin) recorded during the admission were extracted. Clinical characteristics were based upon the Health of the Nation Outcome Scale, a 12-item clinician rated tool contemporaneously rated at admission and discharge.

**Results:**

A total of 968 patients met the inclusion criteria. 309 patients were female and mean age was 38 years. The most frequent ethnicities were White, Black African, Black Other and Black Caribbean and the commonest diagnoses were schizophrenia, unspecified non-organic psychosis and schizoaffective disorder. Mean interval from admission to admission blood test was 0.8 days.

Patients with affective psychosis had a significantly higher white cell count, monocyte count and lymphocyte count than patients with non-affective psychosis on admission. Furthermore, among patients with affective psychosis, a partial correlation controlling for age, body mass index, blood pressure, physical health and smoking status found a significant association between symptom severity and monocyte count. There was a highly significant association between both neutrophil count and white cell count with hallucinatory symptoms. There was also a highly significant positive association between C-reactive protein and self-injurious behaviour, replicating recently published findings in smaller samples. There was a significant reduction in overall psychiatric symptoms over the course of admission, which was significantly associated with admission monocyte count. A partial correlation found white cell count and neutrophil count at admission were associated with reductions in hallucinatory symptoms. Eosinophil count was significantly associated with admission length.

**Discussion:**

In a large cohort of patients admitted due to psychotic disorder, pro-inflammatory markers were associated with affective psychosis and overall symptom severity, and predicted admission length and reduction in symptom severity. The study supports an association between immune dysregulation and psychosis. Furthermore, the study highlights the role of routinely and inexpensively measured peripheral inflammatory markers as potential diagnostic and prognostic biomarkers in psychosis.

